# Editorial: Impact and correlation between migration and psychiatric disorders

**DOI:** 10.3389/fpsyt.2025.1622846

**Published:** 2025-07-11

**Authors:** Davide Prestia

**Affiliations:** IRCCS Ospedale Policlinico San Martino, Genova, Italy

**Keywords:** migration, community resilience, psychiatric disorders, anxiety, depression

Forced migration is one of the defining global challenges of our era, with profound repercussions not only for the individuals and families directly involved but also for the host societies tasked with responding. Amidst humanitarian concerns and debates about integration, mental health stands out as a crucial but often overlooked dimension. Refugees and asylum seekers, displaced by conflict, persecution, or structural instability, face a heightened risk of psychological distress at every stage of the migratory experience: before departure, during the migration journey, and after arrival (1).

The contributions in this Research Topic converge around a key message: the mental health outcomes of migrants cannot be disentangled from their social and relational environments. The prevalence of depression, anxiety, and post-traumatic stress disorder (PTSD) is consistently elevated among refugee populations. However, these disorders do not emerge in a vacuum (2, 3). Their expression is modulated by a complex interplay of trauma exposure, individual resilience, social integration, and the broader context of reception (4).

In host countries, post-migration stressors—such as legal precarity, unemployment, discrimination, and housing instability—can be as damaging to mental health as the original trauma that forced people to flee. Moreover, differences in access to care, linguistic barriers, and a lack of culturally attuned services can further exacerbate psychological vulnerability. These findings call for a reframing of migrant mental health: not merely as a clinical issue, but also as a reflection of social justice, inclusion, and systemic responsiveness.

A recurring and unifying theme across the articles is the concept of community resilience. Far from being a vague or purely rhetorical notion, community resilience is operationalized here as the capacity of both migrant and host communities to mobilize resources, sustain functionality, and support well-being in the face of adversity (5). Migrants who perceive their host environment as supportive, inclusive, and responsive report higher levels of subjective well-being and experience a buffering effect against the psychological burden of trauma and displacement.

This insight is particularly salient when mental health is understood as more than just the absence of illness, but as a positive, dynamic state of functioning—one that includes autonomy, agency, interpersonal relationships, and a sense of belonging. In this regard, the perception of being part of a resilient community—one defined by cohesion, access to information, shared values, and mutual aid—emerges as a protective factor that can mitigate the effects of even severe psychopathology.

Importantly, the research (6) also emphasizes the need to move beyond purely individualized models of care, which tend to isolate suffering from its sociopolitical context. Innovative approaches, such as community-based participatory research and peer-led interventions, are not only clinically promising but also politically and ethically grounded. Empowering migrants to participate in the design and delivery of mental health interventions—such as culturally adapted online EMDR therapy—fosters trust, ownership, and culturally sensitive healing practices (7).

Furthermore, these strategies offer practical solutions to structural limitations, such as a scarcity of trained clinicians, geographic dispersion of refugee populations, and resource constraints in healthcare systems. The use of remote platforms, when combined with community engagement and culturally competent facilitation, demonstrates scalability and effectiveness, especially when delivered by professionals who share the lived experience of displacement (8).

Crucially, these insights invite a paradigm shift in both research and policy. Rather than viewing migrants as passive recipients of aid or as isolated patients in need of treatment, the focus must shift to systemic change and collective empowerment (9). This includes fostering inclusive urban planning, investing in intercultural mediation, and removing administrative barriers to care. Policymakers, practitioners, and communities must recognize that promoting mental health among migrants means creating environments in which they can participate, contribute, and flourish (10).

At the same time, attention must be paid to intersectional vulnerabilities, particularly gender-based violence, economic marginalization, and the effects of discrimination. For instance, migrant women often experience specific forms of trauma and face compounded barriers to accessing support. Tailored approaches that recognize these differences are essential in both prevention and treatment. In this sense, gender-sensitive and culturally congruent care models are not optional—they are prerequisites for equitable health systems.

Ultimately, the articles in this Research Topic collectively advocate for a holistic, multidimensional approach to migrant mental health: one that integrates clinical care with community-based strategies, emphasizes resilience over deficits, and values the voices and knowledge of migrants themselves. These perspectives are not merely academic; they offer a roadmap for more humane and effective responses to forced migration that are grounded in dignity, solidarity, and evidence.

In conclusion, as the phenomenon of migration continues to shape the demographic and cultural fabric of societies worldwide, the question is no longer whether mental health should be part of the migration agenda, but how we choose to address it. Investing in community resilience is not a peripheral concern—it is central to the success of integration policies, the sustainability of health systems, and the construction of inclusive democracies. Supporting migrants in rebuilding their lives also means recognizing their right to psychological safety, social belonging, and full participation in the life of the communities they now call home.
